# No significant impact of platelet‐rich plasma on recovery after Achilles tendon surgery: A double‐blind randomized controlled trial

**DOI:** 10.1002/jeo2.70168

**Published:** 2025-02-13

**Authors:** Youichi Yasui, Wataru Miyamoto, Jun Sasahara, Tsukada Keisuke, Maya Kubo, Gen Sasaki, Asako Yamamoto, Hirotaka Kawano

**Affiliations:** ^1^ Department of Orthopaedic Surgery Teikyo University School of Medicine Itabashi Tokyo Japan; ^2^ Department of Radiology Teikyo University School of Medicine Itabashi Tokyo Japan

**Keywords:** Achilles tendon repair, platelet‐rich plasma, post‐operative recovery, randomized controlled trial, side‐locking loop suture

## Abstract

**Purpose:**

Double‐blind, randomized, placebo‐controlled trials evaluating the efficacy and safety of Platelet‐rich plasma (PRP) in the treatment of Achilles tendon rupture (ATR) have been scant. This study examines the therapeutic impact of PRP injection 3 weeks after surgery in middle‐aged males.

**Methods:**

This double‐blind, randomized, placebo‐controlled trial included consecutive ATR patients who satisfied the inclusion criteria and was conducted from 5 September 2018 to 24 June 2021. Three weeks after surgery using the side‐locking loop technique, PRP or saline was injected at the suture site under ultrasound guidance. Evaluations were conducted at predetermined intervals (6, 10, 12, 16 and 24 weeks and 1 and 2 years) after surgery. The primary outcome was the period needed to perform a bilateral heel raise, and the important secondary outcomes were the periods needed to perform a single heel raise and 20 unilateral heel raises, respectively.

**Results:**

There were seven participants in the PRP group and seven in the saline group. Demographically, both groups exhibited comparable characteristics. No complications were reported. At 6 weeks after surgery, all participants achieved bilateral heel raise. The PRP and saline groups averaged 12.3 ± 2.7 and 15.7 ± 5.9 weeks to achieve a single heel raise and 14.3 ± 2.7 and 17.7 ± 4.5 weeks to achieve 20 unilateral heel raises, respectively, with no significant differences between both groups. Moreover, no substantial disparities in clinical scores, period of jogging initiation and magnetic resonance imaging tendon assessments were noted.

**Conclusions:**

PRP did not offer a distinct advantage over saline in terms of recovery from ATR in middle‐aged males. This finding underscores the need to reassess the post‐operative significance of PRP and highlights the importance of further research to determine its potential advantages and risks.

**Level of Evidence:**

Level I.

**Abbreviations**
ATR: Achilles tendon rupture
ATRS: Achilles tendon total rupture score
EGF: epidermal growth factor
JSSF: Japanese Society for Surgery of the Foot
MRI: magnetic resonance imaging
PRP: platelet‐rich plasma
ROI: region of interest
SLLS: side‐locking loop suture

## INTRODUCTION

Achilles tendon rupture (ATR), which affects both elite and recreational athletes, is particularly prevalent in middle‐aged males, especially those participating in sports activities [18, 36]. This demographic is at the highest risk for ATR, with the greatest incidence occurring in men aged 40–60 years [17, 18]. Current debates focus on comparing the effectiveness of surgical and non‐surgical treatments, a factor that considerably affects clinical decision‐making [29, 30]. Despite various treatments, the return‐to‐play ratio for athletes often extends beyond 5 months after injury, with an approximate return‐to‐play rate of 80% [13, 36]. Muscle strength is a critical factor in recovery, as deficits in calf muscle strength and endurance can persist for years after an ATR, leading to reduced physical performance and impacting activities of daily living [36]. These statistics emphasize the need for innovations that expedite recovery and boost success, promising superior patient outcomes [13].

In this dynamic therapeutic landscape, platelet‐rich plasma (PRP) has gained traction as a potential supplementary treatment. PRP, obtained by centrifuging whole blood, is replete with platelets known for their regenerative capacities [2]. Basic science studies suggest PRP strengthens the biomechanical integrity of healing tendons, potentially enhancing functional results and shortening recovery [2, 12]. However, despite its potential, there have been no double‐blind, randomized, placebo‐controlled trials evaluating the efficacy of PRP in ATR repair, leaving a significant gap in the clinical evidence.

The current double‐blind, randomized, placebo‐controlled trial aimed to evaluate the therapeutic efficacy and safety profile of PRP injection administered 3 weeks post‐surgery at the suture locus after ATR repair. This time point was selected to ensure that the surgical wound had healed sufficiently, reducing the risk of PRP leakage and maximizing its potential therapeutic effect. The hypothesis of the current study was that post‐operative injection of a single PRP injection would enhance recovery. The primary outcome, the ability to perform a bilateral heel raise, was central to this evaluation. By evaluating the effects of PRP on muscle strength recovery in middle‐aged males, who are the most susceptible to ATR, the study contributes valuable insights and highlights the significance of targeted treatment strategies in this high‐risk population.

## METHODS

### Study design

The current study adopted a randomized, double‐blind, placebo‐controlled trial design and was conducted at the institution from 5 September 2018 to 24 June 2021. The full 2‐year follow‐up had been completed by 15 July 2023. Throughout the trial period, both the outcome assessors and participants were unaware of the group allocations. This trial had been officially registered in the country (registration number: UMIN000036496, https://center6.umin.ac.jp/cgi-open-bin/ctr/ctr.cgi?function=brows&action=brows&recptno=R000041585&type=summary&language=J). Moreover, ethics approval was obtained by the government and local association committee (CONCIDE Certified Regenerative Medicine Committee, approval No. CNCD3‐30001) and the ethical standards of the institutional review board of Teikyo University (approval number: 17‐165‐2) and conformed with the ethical principles outlined in the Declaration of Helsinki. To ensure the validity of the present study, an external research management institute in the authors' country monitored the entirety of the study. The CONSORT checklist was utilized during the writing process.

Details regarding the study timeline are presented in Table 1. Two seasoned physicians, each with over 15 years of experience in foot and ankle surgery, exclusively managed the diagnosis, surgical interventions and post‐operative care. A week following Achilles tendon suturing, blood samples for PRP preparation were collected from all participants. Three weeks after surgery, either PRP or saline was administered at the suture site. Evaluations were systematically conducted at predefined intervals.

**Table 1:** Timeline of the present study.

		0 w	1 w	3 w	6 w	10 w	12 w	16 w	24 w	1 y	2 y
Preoperative evaluation	〇										
Informed consent	〇										
Register	〇										
Surgery		〇									
The collection of blood samples			〇								
Allocation			〇								
PRP or saline injection				〇							
Heel raise					〇	〇	〇	〇	〇	〇	〇
Initiation of jogging						〇	〇	〇	〇	〇	〇
Clinical scores; JSSF※1	〇				〇	〇	〇	〇	〇	〇	〇
Clinical scores; ATRS※2	〇				〇	〇	〇	〇	〇	〇	〇
Gastrocnemius muscle strength	〇※3								〇※4	〇※4	〇※4
MRI							〇		〇	〇	〇

*Note*: ※1 JSSF: Japanese Society for Surgery of the Foot, #2 ATRS: Achilles tendon total rupture score, ※3 Non‐injured side, #4 Bilateral sides.

Abbreviations: MRI, magnetic resonance imaging; PRP, platelet‐rich plasma; w, weeks; y, years.

### Participants

Initial candidates were individuals diagnosed with ATR at the orthopaedic outpatient department. Diagnoses were established based on medical interviews, physical examinations and magnetic resonance imaging (MRI) assessments. Although the experienced physicians were likely to accurately diagnose ATR, physical examination alone has a sensitivity ranging from 73% to 96% and a specificity from 85% to 93% [1]. Ultrasound, although useful, has a sensitivity of 94.8% and a specificity of 98.7% [1]. To ensure the highest level of diagnostic certainty and meet the requirements of the external research management institute overseeing the study, MRI was chosen to confirm the diagnosis of ATR. Inclusion criteria included the following: male, age 30–59 years, MRI‐confirmed ATR at the tendinous portion, unilateral injuries at the initial site, preference for surgery, and written informed consent. Exclusion criteria comprised smoking or presence of conditions like diabetes, tumours or inflammatory joint diseases (e.g., rheumatoid arthritis). Additionally, patients with a history of injuries or fractures in both feet and ankles were excluded. Physicians also considered excluding candidates who, although meeting the medical criteria, were deemed unlikely to complete the study due to personal circumstances such as frequent relocations or other factors affecting long‐term participation. These selection criteria ensured a homogeneous study group for focused investigation.

Prospective participants who satisfied the explicit eligibility criteria were briefed about the study nuances by the physicians. They received comprehensive information on the potential risks associated with the study before providing their written informed consent.

### Operative technique and post‐operative protocol

The surgical intervention was conducted under general anaesthesia, with the patient oriented in a prone position. A longitudinal skin incision was created slightly medial to the central line of the Achilles tendon to expose the rupture site. The Achilles tendon was meticulously repaired employing the side‐locking loop suture (SLLS) technique utilizing a USP number 5 braided polyblend suture (FiberWire; Arthrex Inc) [20, 32]. During the procedure, tension was applied to both ends of the tendon while ensuring congruent alignment through knee flexion to 90° and neutral dorsiflexion of the ankle. This technique offers high tensile strength and allows for significantly faster post‐operative rehabilitation. It enables early mobilization without the need for prolonged immobilization [11, 20, 32].

In the post‐operative phase, the limb was immobilized using a below‐knee splint for 3 days. Subsequently, patients initiated partial weight‐bearing manoeuvres without any orthosis and began active range‐of‐motion exercises based on their pain tolerance levels. Two weeks after surgery, the sutures were removed, followed by the administration of either PRP or saline injections without further rest periods. From the fourth week onwards, patients progressed to full weight‐bearing without crutches and started double‐leg heel raise exercises based on individual pain thresholds. At 8 weeks after surgery, those capable of executing double‐leg heel raises advanced to jogging and single‐leg heel raise exercises. Patients were allowed to jog within their comfort range, accommodating individual variations. Specific parameters such as time, distance, or speed for jogging were not strictly defined. Thereafter, the rehabilitation plan included a graded reintroduction to athletic activities, monitored meticulously to prevent any potential setbacks, with the benchmark being the successful completion of 20 consecutive single‐leg heel raises.

### Preparation and quality control of the PRP

One week after surgery, blood samples essential for PRP preparation were collected from all participants and underwent strict processing through a well‐established and standardized protocol. The PRP utilized in this research was exclusively sourced from FUJISOFT Tissue Engineering Co., Ltd, an entity holding requisite accreditation in the country (Facility Number: FA3150002). The formulation used set a benchmark for reproducible content concentration, as validated by a previous study [16]. This unique formulation, a novel development within the country, has been rigorously analyzed and evaluated in a university hospital environment. Additionally, it has been subjected to strict sterility tests following national regulatory guidelines, thereby confirming its safety profile. Prior studies, including a research study by Fujiwara in 2016, emphasize that the epidermal growth factor (EGF), a vital agent in tissue repair, maintains a stable concentration for up to 3 months after purification under optimal conditions below 4°C [9]. To guarantee superior quality, meticulous measurements were conducted to ascertain the platelet concentration and EGF content within the PRP.

In accordance with the principles of a double‐blind study, the prepared PRP was securely stored in opaque vials to uphold the blinding protocol, while stringent quality control measures and coloured injection syringes were employed to prevent content identification from external observation.

### Intervention

Following the collection of blood samples 1 week after surgery, all patients were allocated to the PRP or saline group. This allocation marked the initiation of the double‐blind trial, which was managed by a central clinical research support centre utilizing a centralized randomization method. Group assignments were not revealed to the statistician until the comprehensive 2‐year follow‐up encompassing all case studies had concluded.

At 3 weeks after surgery, patients were set to receive either PRP or saline at the sutured tendon site. This specific time point was chosen because it is generally understood that the surgical wound is fully healed by this stage, minimizing the risk of PRP leakage from the site and optimizing its therapeutic efficacy. At the time of injection, the surgical wound had indeed healed in all patients. The protocol necessitated placing patients in the prone position, introducing local anaesthesia (1% lidocaine, 1.0 mL) and executing precise ultrasound‐guided injections of either PRP or saline (1.5 mL) at or potentially inside the ATR site. An adept orthopaedic specialist with substantial experience in ultrasound‐guided injections performed this critical procedure and remained uninvolved in subsequent evaluations. Additionally, the evaluator was deliberately absent during the administration phase, preserving the integrity of the double‐blind study design.

To further maintain the blinding criteria, the syringe used for the injection was distinctly coloured, preventing any external identification of its contents.

### Evaluation criteria

As outlined in Table 1, this study utilized a detailed set of evaluation criteria vital for assessing the efficacy of the therapy, which comprised the following elements:

Primary outcome

- The period needed to execute one bilateral heel raise
  This key metric, which had been considered a significant indicator of recovery progression, gauges the duration participants needed to execute one repetition of bilateral heel raise [33]. To ensure accurate assessment of the bilateral heel raise, 2 cm high blocks were placed under both heels, and it was confirmed that both sides could exceed this height. Initially, to assess asymmetry in muscle recovery, scales were used under both feet to monitor weight distribution during the heel raises. However, after observing that providing proper instructions to balance weight was sufficient to achieve equal distribution, and due to a patient tripping on the scale, the use of scales was discontinued for safety reasons.
  Secondary outcomes
- The period needed to execute 1 and 20 repetitions of a single heel raise.
  This metric denotes the duration within which participants completed 1 and 20 repetitions of single heel raises, illustrating the recovery trajectory of the injured limb [23].
- Clinical scores; Japanese Society for Surgery of the Foot (JSSF) [21] and Achilles tendon total rupture score (ATRS) [22].
- Commencement of jogging.
- MRI analysis of the ruptured tendon.

MRI was performed using a 3‐T scanner (Skyra, Siemens Healthineers) with a commercially available 16‐channel ankle coil. MRI scans were conducted at multiple time points, including 12 weeks, 24 weeks, 1 year and 2 years post‐operatively (Table 1). Accordingly, axial T2‐weighted images were obtained using the following parameters: repetition time/echo time, 700/15 ms; slice thickness, 3.0 mm; field of view, 16 cm; matrix size, 448 × 336; flip angle, 30°; number of excitations, 1; 27 slices above the lowest attachment site of the Achilles tendon to the calcaneus.

All cases were reviewed on a clinical workstation (Ziostation2, Ziosoft) and vertical, horizontal diameters (mm) and the area (mm^2^) were measured at the centre of the sutured area. Signal intensity was also measured manually using the region of interest (ROI) measurement tool for the Achilles tendon and flexor digitorum longus muscle tendon with the same slice, after which the average signal intensities were recorded. The signal intensity ratio was calculated using the following formula: average signal intensity of the Achilles tendon/average signal intensity of the flexor digitorum longus muscle tendon.

T2 quantification sequences were obtained using a sagittal turbo‐spin echo multi‐echo acquisition with the following settings: repetition time/echo time, 1300/5 echo times (9.2, 18.4, 27.6, 36.8 and 46 ms); field of view, 20 cm; matrix size, 320 × 320; flip angle, 180°; number of excitations, 1; 3 slices at the centre of the tendon [10]. The Achilles tendon margin was manually enclosed in the polygonal ROI for T2 value calculation. The upper and lower ends of the margin were set according to the sutured thread. These measurements were obtained by one musculoskeletal radiologist with 15 years of experience who was blinded to the treatment assignment.

### Sample size calculation

This study aimed to include 14 participants throughout the designated research duration who were to be segregated into two groups, each containing 7 individuals, within a rigorously structured double‐blind trial. The requisite sample size was determined through analytical insights derived from relevant literature [25]. This groundwork revolves around the projected timeframe from Achilles tendon surgery to the achievement of a single bilateral heel raise, the primary evaluation metric. As projected, the duration was roughly 11 and 18 weeks for individuals in the PRP and control groups, respectively, assuming a standard deviation of 3 weeks. Utilizing a two‐tailed *t* test for preliminary computations, a group size of 6 was deemed sufficient to achieve a statistical power of 90%, with a 5% significance level. However, given the intention to employ the Mann–Whitney *U* test in the final analysis, which might marginally decrease detection power, the sample size was prudently expanded to 7 per group to enhance the reliability and precision of the study.

### Statistical analysis

Statistical analysis was conducted by an experienced, independent, and blinded biostatistician. Continuous data were presented as mean (SD) or median (interquartile range, IQR). Temporal changes in each evaluation item for both groups were analyzed using repeated measures analysis of variance. For individual evaluations in both groups, continuous data were evaluated using the unequal variances *t* test and Wilcoxon rank‐sum test. Statistical analyses were performed in July 2023 using the R Project for Statistical Computing (version 4.3.0).

## RESULTS

### Participant demographics

None of the participants were excluded based on the eligibility criteria established prior to the study (Flow chart 1). Initially, the study enlisted 17 participants. Unfortunately, three individuals had to withdraw due to injection phobia, PRP preparation scheduling conflicts, and unsuccessful PRP purification. The remaining participants were equally distributed into two groups: the PRP group and the saline group, each consisting of seven patients (Table 2).

**Flow Chart 1:**
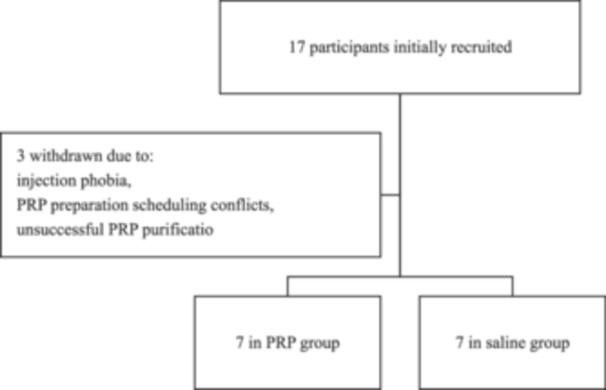
Flow chart of inclusion and exclusion process during the study.

**Table 2:** Patient demographic data and the outcome of the primary outcome.

	Patient	Age (years)	BMI	Side	Preoperative sport	Period from the injury to surgery (days)	Platelet in blood (10^4^/µL)	Platelet in PRP (10^4^/µL)	EGF (pg/mL)	Period needed to execute one bilateral heel raise (weeks)
PRP	1	50	24.9	Right	Badminton	16	25.3	177	3426	6
	2	31	28.7	Right	Baseball	8	29.6	205	4086	6
	3	59	27.7	Left	Jiujitsu	16	30.9	140	2557	6
	4	46	34.0	Left	Volleyball	9	37.0	264	5963	6
	5	36	23.5	Left	Futsal	7	22.9	150	2933	6
	6	32	21.1	Left	Basketball	12	14.8	81.0	1217	6
	7	33	29.3	Right	Jogging	17	25.9	132	3335	6
Control	1	45	26.7	Right	Badminton	8	N/A	N/A	N/A	6
	2	35	24.0	Left	Futsal	8	N/A	N/A	N/A	6
	3	35	35.5	Left	Jiujitsu	12	N/A	N/A	N/A	6
	4	32	23.6	Left	Futsal	7	N/A	N/A	N/A	6
	5	48	23.3	Right	Boxing	8	N/A	N/A	N/A	6
	6	36	26.7	Left	Futsal	11	N/A	N/A	N/A	6
	7	36	23.3	Left	Tennis	8	N/A	N/A	N/A	6

Abbreviations: BMI, body mass index; EGF, epidermal growth factor; PRP, platelet‐rich plasma.

Throughout the study duration, no perioperative and injection‐related complications were noted. No notable disparities in participant attributes, such as age and sex, the period from the injury to surgery were observed. Furthermore, the PRP utilized in this study exhibited a significantly higher platelet concentration, surpassing standard blood platelet levels, thereby affirming the efficacy of PRP preparation [31].

### Primary outcomes

At the 6‐week post‐operative evaluation, both groups were able to execute a single repetition of bilateral heel raise. No statistically significant difference was observed between the two groups (Table 2).

### Secondary outcomes

The analysis indicated no statistically significant difference in the timing at which the PRP (12.3 ± 2.7 weeks) and saline groups (15.7 ± 5.9 weeks) accomplished one repetition of a single heel raise. At the 12th week of post‐operative evaluation, a substantial percentage of the patients from both cohorts (five out of seven in the PRP group and four out of seven in the saline group) were able to perform a single heel raise, indicating no statistically significant difference in the rehabilitative trajectories between the groups. The PRP and saline groups required 14.3 ± 2.7 and 17.7 ± 4.5 weeks to achieve 20 repetitions of single heel raises, respectively, the difference between which was not statistically significant.

No statistically significant in the JSSF and ATRS scores were noted between the groups (Figures Chart 1, 1 and 2). Data regarding the commencement of jogging activities demonstrated a comparable time frame between both groups (8.1 ± 0.3 vs. 8.0 ± 0 weeks in the PRP and saline groups, respectively), with no statistically significant difference.

**Figure 1:**
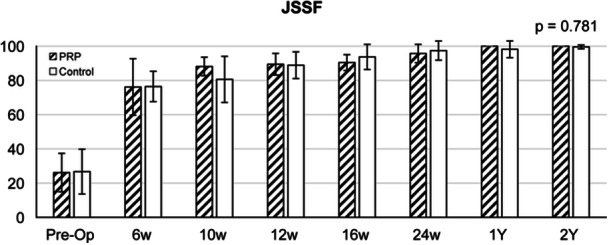
Japanese Society for Surgery of the Foot score. ATRS, Achilles tendon total rupture score; PRP, platelet‐rich plasma.

**Figure 2:**
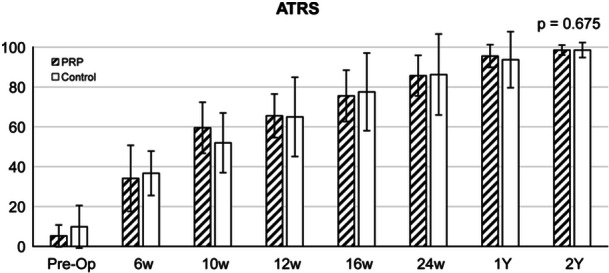
Achilles tendon total rupture score. PRP, platelet‐rich plasma.

**Figure 3:**
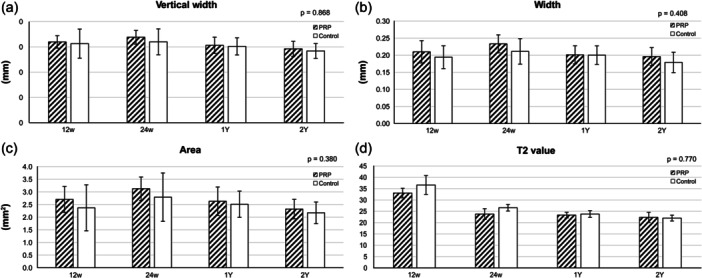
Results of MRI analysis. (a) Vertical width. (b) Width. (c) Area. (d) T2 value. MRI, magnetic resonance imaging.

**Figure 4:**
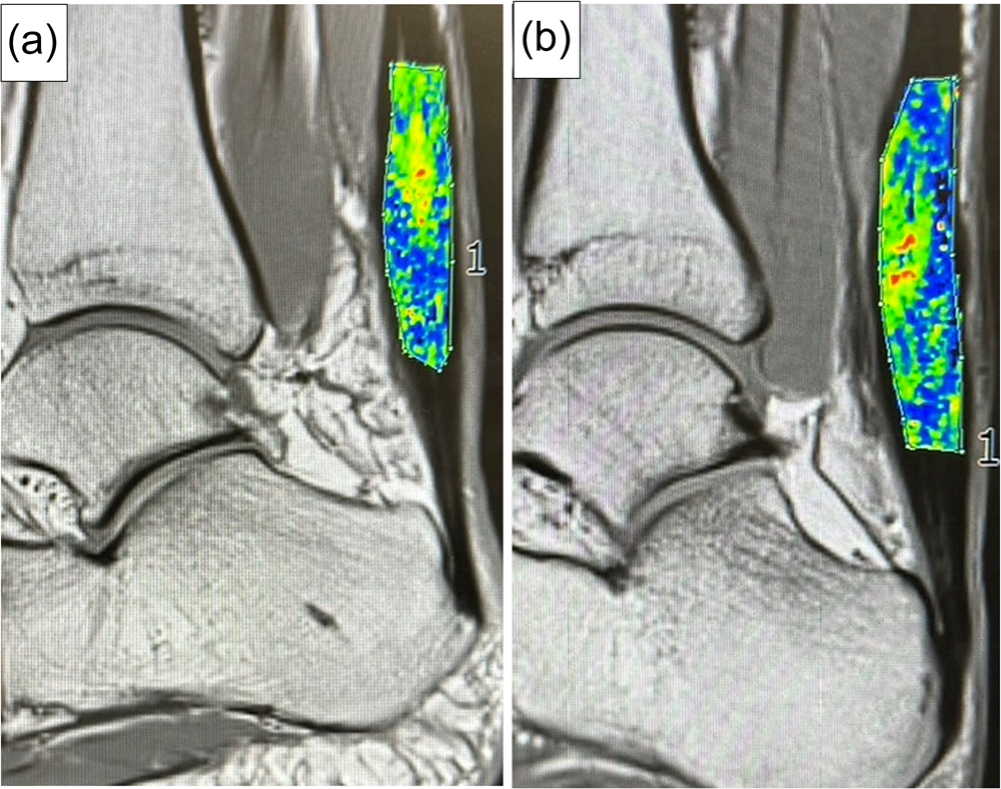
This figure presents representative cases from the PRP (A: patient no. 7) and saline groups (B: patient no. 7) at the 2‐year time point, highlighting the T2 mapping results. The selected images provide a clear comparison of the healing process between the two treatment groups. PRP, platelet‐rich plasma.

Moreover, MRI conducted to assess various attributes, including the width, area and T2 mapping values, corroborated the primary outcomes, highlighting no statistically significant difference in the evaluated parameters between the PRP and saline groups (Figures 3 and 4).

## DISCUSSION

The most important finding of this randomized, double‐blind, placebo‐controlled trial was that the injection of PRP after ATR surgery, particularly 3 weeks thereafter, did not demonstrate any significant benefit over placebo. Based on the previous literature, the theoretical benefits attributed to PRP, such as enhanced tendon healing and accelerated muscle recovery, are noteworthy [2]. However, no significant improvements were observed in clinical scores, muscular strength metrics, or radiographic evaluations. This study fills a critical gap in the existing literature, being the first double‐blind randomized placebo‐controlled trial to evaluate the efficacy of PRP in the context of ATR repair, particularly in middle‐aged males. Further studies are needed to determine whether these findings can be generalized to other populations, such as younger athletes, females, and older patients.

While the current study focuses on the clinical outcomes of PRP use in ATR repair, it is important to note the unique anatomical challenges that influence tendon healing. The Achilles tendon, being a bradytrophic and hypovascular tissue, inherently presents healing difficulties, especially in its mid‐portion, which receives the least blood supply [27]. Furthermore, this region of the tendon is prone to adhesion and scarring, which can limit tendon mobility during the recovery phase [27]. These anatomical factors may further explain the challenges of achieving early recovery.

A systematic review conducted by Baksh et al. examined basic scientific literature on the use of PRP in tendon models, reported several potential effects of PRP on tendon models compared to a control and suggested that establishing a proof of concept for PRP might lead to more high‐quality clinical studies to define its appropriate indications [2]. Some studies using animal models have expressed scepticism regarding the effectiveness of ATR [5, 24, 28]. In fact, Parafioriti et al., who performed histological and immunohistochemical examinations in a rat Achilles tendon tear model, found that a single local PRP injection provided no benefit [24]. Moreover, Çirci et al. conducted histological and biomechanical examinations in a rat Achilles tendon tear model to determine the optimal timing for PRP injection. They compared two injection timings: immediately after Achilles tendon transection and 3 days thereafter. Notably, no significant differences in all examinations were observed between the control and experimental groups regardless of timing [5]. It is worth noting that the mentioned studies used donor rats to produce PRP given the technical difficulty of creating autologous PRP in a rat model. In an attempt to use autologous PRP in an animal study, Şen et al. applied rabbit ATR models. However, despite hypothesizing that a single PRP injection would be effective, their histopathological and biomechanical evaluations showed that PRP had no impact on the healing process 28 days after ATR [28]. While PRP has shown promise in basic science studies, a 2022 review highlights that the breadth and depth of evidence delineating the influence of PRP on ATR surgical outcomes remain inconsistent [4, 6, 7, 25, 26, 34, 37].

Regarding conservative treatment, a retrospective comparative study by Kaniki et al., which investigated the effectiveness of two PRP injections for non‐surgical ATR (i.e. first within 2 weeks after injury and second 2 weeks after first injection), found no measurable benefits to the addition in isokinetic strength for plantar flexion, ankle range of motion (ROM), leg circumference, and clinical scores including the Leppilahti and The American Orthopaedic Foot & Ankle Society scores [14]. This sentiment has been echoed in more recent studies [3, 15]. The recurring theme across these studies was the limited efficacy of PRP in non‐surgical ATR treatment modalities. One proposed hypothesis for these consistent findings was the potential delay in implementing ROM exercises after treatment, a factor that has been underscored for its importance in facilitating tendon healing. It is well known that stimulation of the ruptured portion of the tendon through ROM exercise facilitates tendon healing [27]. These findings underscore the limited efficacy of PRP in non‐surgical treatment, which contrasts with its potential role in surgical applications, where early ROM exercises are often initiated sooner.

Two pivotal prospective randomized controlled trials have elucidated the efficacy of adjunctive PRP therapy after ATR surgical procedures [26, 37]. In 2011, Schepull et al. reported the results of their randomized controlled trial, in which a single PRP was injected intraoperatively into the allocated PRP patients, while no injections were given to control patients [26]. This study included 30 participants (16 in the PRP group and 14 in the control group). Their results revealed no discernable benefits of PRP based on comprehensive radiological and clinical metrics assessed within one post‐operative year. Conversely, a 2016 study by Zou et al. documented improvements in multiple parameters after surgery in the PRP cohort [37]. In this study, there were 36 participants (16 in the PRP group and 20 in the control group). Although the injection timing of PRP was same as that utilized in the study by Schepull et al., the results showed that isokinetic muscle parameters at 3 months, SF‐36 and Leppilahti scores at 6 and 12 months and ROM at 6, 12 and 24 months after surgery were better in the PRP group than in the control group, suggesting the safety and efficacy of PRP as a biological augmentation agent for surgical repair of ATR. Although both studies had the same timing of PRP injections, their post‐operative rehabilitation protocol varied widely. Schepull et al. applied cast immobilization for 7 weeks after surgery, whereas Zou et al. applied a splint for 3 weeks and initiated active ROM exercise promptly thereafter. Notably, variations in post‐operative rehabilitation protocols between the studies may account for discrepancies in outcomes.

To assess functional recovery, this study selected bilateral heel raises as the primary outcome based on several key considerations related to both clinical relevance and the methodological challenges of conducting a double‐blind, randomized, placebo‐controlled trial on PRP in ATR repair. First, no previous double‐blind randomized placebo‐controlled trial has been conducted on the use of PRP for ATR, leaving a significant gap in the literature. This study aims to fill that gap by evaluating the role of PRP in enhancing muscle recovery, particularly in middle‐aged males, who are highly susceptible to ATR. Second, the SLLS technique used in this study consistently yields near‐perfect early post‐operative clinical outcomes, as demonstrated in previous studies [11, 20, 32]. This often leads to a ceiling effect, where traditional clinical scores fail to capture subtle differences in recovery. For this reason, calf muscle strength was chosen as a more sensitive functional outcome measure and an early, observable indicator of tendon recovery and muscle strength [20, 33]. Calf muscle strength can be assessed using either heel raises or devices such as the Biodex dynamometer. However, dynamometers are not commonly used in routine post‐operative evaluations due to limited accessibility. Among the heel raises, single‐leg heel raises are known to closely correlate with favourable clinical outcomes [23]. However, bilateral heel raises tend to recover earlier, making them a more feasible and an early marker of functional recovery. Based on prior research indicating a faster return to activities like jogging in PRP‐treated patients [25], Bilateral heel raises were hypothesized to similarly reveal early differences in recovery between the PRP and control groups. This assumption allowed for the estimation of a 7‐week difference between the groups in the time to achieve bilateral heel raises, which informed the sample size calculation.

An external research management entity in the authors' country monitored the entirety of the study, validating its content and outcomes. The present study explored the combined efficacy of PRP with the modified SLLS technique, which allows early post‐operative rehabilitation [20]. The SLLS technique, known for its high tensile strength, allows early mobilization and faster rehabilitation, enabling patients to begin weight‐bearing exercises shortly after surgery [11, 20, 32]. This technique reduces the need for prolonged immobilization and supports an earlier return to daily activities and sports [11, 20, 32]. Rehabilitative exercises were initiated 3 days after surgery, considering prior evidence suggesting that early ROM exercises can enhance tendon healing [11, 20, 27, 35]. PRP administration was strategically delayed until 3 weeks post‐surgery to mitigate leakage concerns observed in clinical practice and described in a previous study [26]. By this time, the wound had fully healed, minimizing the risk of PRP leakage. In contrast, previous studies have reported concerns about PRP leakage when injected earlier, which may have reduced its efficacy [26]. This discrepancy highlights the importance of selecting the optimal timing for PRP injections to maximize their therapeutic benefits. Despite anticipations of early ROM exercises and the effectiveness of PRP, the comparison between the PRP and control groups revealed that PRP provided no marked benefit in post‐operative recovery. Regarding the period of the jogging, the data shows that some patients were able to start jogging before they could complete 20 repetitions of single‐leg heel raises. This might seem confusing as calf muscle strength is often associated with functional recovery. However, jogging involves a broader range of physical coordination beyond just calf muscle strength, including the use of the trunk and upper limbs. For example, in daily clinical practice, patients with fixed ankle joints can still jog by employing compensatory strategies and adaptations [8].

The primary limitation of the current research is its limited sample size. Historically, SLLS patients have demonstrated near‐perfect clinical scores at the final follow‐up [20, 35], resulting in minimal score variations. Therefore, the study shifted its emphasis toward gastrocnemius muscle function recovery. In the absence of similar randomized placebo‐controlled trials, the metrics from Sanchez's study were adopted [25]. While a power analysis was performed to ensure 90% power to detect a clinically meaningful difference, assuming a two‐tailed *α* of 0.05, the constraints imposed by the COVID‐19 pandemic hindered recruitment efforts. This may have impacted the study's power to detect smaller differences. Although the sample size was adequate for the primary outcome, the exploratory nature of secondary outcomes and the reduced sample size may have contributed to the lack of significant findings in those metrics. Notably, one repetition of a single heel raise revealed a divergence of 1 month, hinting at the possibility of a type II error, especially in the secondary outcomes, which were not specifically powered for significance testing. In randomized placebo‐controlled trials, secondary outcomes are often exploratory and may not be powered to detect significant differences, unlike the primary outcome, which is used to determine sample size and draw conclusions. Therefore, the non‐significant findings in secondary outcomes reflect their exploratory nature and the potential for type II errors. PRP leucocyte and growth factor components remained unexamined due to budgetary constraints. Recent basic scientific studies published in 2022 highlight the potential role of PRP leucocytes in tendon healing [19]. Another limitation of the study was the inclusion of solely middle‐aged males given their susceptibility to ATR. This limitation affects the external validity of the results, as the findings may not be generalizable to other age groups or females. Further research is required to assess the efficacy of PRP in a broader population, including other age groups and female patients, to determine whether these results can be applied beyond the current cohort. Regarding the timing of PRP injection, the 3‐week post‐operative window was chosen because at this time, the surgical wound is typically healed, minimizing the risk of PRP leakage from the suture site and potentially maximizing its therapeutic effect. However, the optimal timing for PRP injections remains uncertain, and this choice may represent a limitation of the study. These limitations highlight the importance of further research to determine the potential advantages and consequences associated with PRP.

A notable limitation of the present study was the inability to conduct a standardized cost analysis for PRP therapy. The cost of PRP varies significantly across countries and institutions, with no standardized pricing model due to the lack of public insurance coverage in many regions. Furthermore, the manufacturing costs of PRP are not transparently disclosed, making it difficult to provide a comprehensive cost evaluation. Given these factors, and the absence of demonstrated clinical benefits in the current study, a cost analysis was not included, as it would not yield meaningful conclusions regarding the cost‐effectiveness of PRP in this context. However, even if PRP therapy were proven to be clinically effective, its widespread adoption would be contingent upon its economic feasibility. Without a clear understanding of the cost‐effectiveness of PRP therapy, especially in comparison to other treatment options, it remains challenging to justify its routine use. Therefore, a more thorough economic evaluation, considering both clinical outcomes and cost, is essential for guiding future clinical decisions and healthcare policies.

## CONCLUSIONS

This study demonstrated that the injection of PRP did not result in superior recovery outcomes compared to saline based on the study parameters. This indicates that PRP does not seem to add any benefit to surgically treated traumatic ATRs in middle‐aged males. Given these outcomes, there is a pressing need to critically evaluate the post‐operative utilization of PRP. Further comprehensive studies are imperative to ascertain the broader clinical implications and potential advantages of PRP considering the limitations of this study.

## AUTHOR CONTRIBUTIONS

Youichi Yasui and Wataru Miyamoto contributed to the conceptualization and design of the study. They were also involved in patient care, data collection, drafting the original manuscript and subsequent reviewing and editing. Jun Sasahara administered the ultrasound‐guided injections and also participated in the editing process. Tsukada Keisuke was in charge of data analysis and took part in both writing and editing the draft. Maya Kubo and Gen Sasaki were responsible for collecting blood samples and reviewing and editing the manuscript. Asako Yamamoto played a role in the conceptualization of MRI analysis, evaluated the MRI results, and contributed to the writing and editing of the draft. Hirotaka Kawano participated in the conceptualization, study design and the review and editing processes.

## CONFLICT OF INTEREST STATEMENT

The authors declare no conflicts of interest.

## ETHICS STATEMENT

This study received ethics approval from the CONCIDE Certified Regenerative Medicine Committee (approval number NB3150043) and the local institutions (17‐165, Teikyo University Ethical Review Board for Medical and Health Research Involving Human Subjects). Written informed consent was obtained from the participants for inclusion in the study.

## Data Availability

The data sets used and/or analyzed during the current study are available from the corresponding author upon reasonable request.
